# Emerging Role of [^18^F]FLT PET/CT in Lymphoid Malignancies: A Review of Clinical Results

**DOI:** 10.3390/hematolrep16010004

**Published:** 2024-01-11

**Authors:** Anna Giulia Nappi, Giulia Santo, Lorenzo Jonghi-Lavarini, Alberto Miceli, Achille Lazzarato, Flavia La Torre, Francesco Dondi, Joana Gorica

**Affiliations:** 1 Section of Nuclear Medicine, Interdisciplinary Department of Medicine, University of Bari “Aldo Moro”, Piazza Giulio Cesare 11, 70124 Bari, Italy; anna.giulia.nappi@gmail.com; 2 Department of Experimental and Clinical Medicine, “Magna Graecia” University of Catanzaro, 88100 Catanzato, Italy; giulia.santo@unicz.it; 3 Nuclear Medicine Department, IRRCS San Gerardo Dei Tintori Di Monza, 20900 Monza, Italy; l.jonghilavarini@campus.unimib.it; 4 Nuclear Medicine Unit, Azienda Ospedaliera SS. Antonio E Biagio E Cesare Arrigo, 15121 Alessandria, Italy; alberto.miceli@ospedale.al.it; 5 Nuclear Medicine Unit, ARNAS G. Brotzu, 09047 Cagliari, Italy; achille.lazzarato@aob.it; 6 Nuclear Medicine Unit, Department of Biomedical and Dental Sciences and of Morpho Functional Imaging, University of Messina, 98125 Messina, Italy; latorreflavia@gmail.com; 7 Nuclear Medicine, ASST Spedali Civili Di Brescia and Università degli Studi di Brescia, 25123 Brescia, Italy; 8 Department of Radiological Sciences, Oncology and Anatomo-Pathology, Sapienza, University of Rome, 00161 Rome, Italy; joana.gorica@uniroma1.it

**Keywords:** hematopoietic malignancies, hematology, positron emission tomography, PET/CT, [^18^F]FLT PET/CT, 3′-deoxy-3′-[^18^F]fluorothymidine, FLT

## Abstract

Fluorine-18 fluorodeoxyglucose ([^18^F]FDG) is nowadays the leading positron emission tomography (PET) tracer for routine clinical work-ups in hematological malignancies; however, it is limited by false positive findings. Notably, false positives can occur in inflammatory and infective cases or in necrotic tumors that are infiltrated by macrophages and other inflammatory cells. In this context, 3′-deoxy-3′-[^18^F]fluorothymidine ([^18^F]FLT) has been shown to be a promising imaging biomarker of hematological malignant cell proliferation. In this review, a total of 15 papers were reviewed to collect literature data regarding the clinical application of [^18^F]FLT PET/CT in hematological malignancies. This imaging modality seems to be a suitable tool for noninvasive assessment of tumor grading, also showing a correlation with Ki-67 immunostaining. Moreover, [^18^F]FLT PET/CT demonstrated high sensitivity in detecting aggressive lymphoma lesions, especially when applying a standardized uptake value (SUV) cutoff of 3. At baseline, the potential of [^18^F]FLT imaging as a predictive tool is demonstrated by the low tracer uptake in patients with a complete response. However, its use is limited in evaluating bone diseases due to its high physiological uptake in bone marrow. Interim [^18^F]FLT PET/CT (iFLT) has the potential to identify high-risk patients with greater precision than [^18^F]FDG PET/CT, optimizing risk-adapted therapy strategies. Moreover, [^18^F]FLT uptake showed a greater ability to differentiate tumor from inflammation compared to [^18^F]FDG, allowing the reduction of false-positive findings and making the first one a more selective tracer. Finally, FLT emerges as a superior independent predictor of PFS and OS compared to FDG and ensures a reliable early response assessment with greater accuracy and predictive value.

## 1. Introduction

Hematological malignancies affect a significant number of patients worldwide each year, for which timely diagnosis and treatment are crucial [1,2]. Although fluorine-18 fluorodeoxyglucose ([^18^F]FDG) remains the leading positron emission tomography (PET) tracer for routine clinical work-up in many neoplasms and in such hematological malignancies, there is an increasing demand for more specific tracers targeting other metabolic pathways to guide early effective treatment strategies [3,4,5]. In this setting, the role of this imaging modality for the initial assessment, post-therapeutic evaluation, and prognostic value has been clearly demonstrated for both Hodgkin lymphoma (HL) and non-HL (NHL), such as diffuse large B-cell lymphoma (DLBCL) or follicular lymphoma [6,7]. As known, false-positive findings in [^18^F]FDG PET/CT are frequent in specific inflammatory and infective cases such as granulomatous disease, sarcoidosis, brown adipose tissue activation, and rebound of thymic hyperplasia. Additionally, false positives can occur in necrotic tumors that are infiltrated by macrophages and other inflammatory cells with heightened glucose metabolism [8,9]. Nevertheless, achieving accurate initial staging, early response evaluation, and follow-up assessments remains essential to optimizing the management of patients with hematological malignancies [8].

One of the possible strategies to enhance specificity in these cases might be the employment of radiopharmaceuticals capable of measuring tumor growth and DNA synthesis [10]. [^11^C]thymidine was the first PET tracer used for noninvasive imaging of tumor proliferation, but its short half-life and rapid metabolism in vivo made it less suitable for routine use in clinical practice [11]. More recently, the thymidine analogue 3′-deoxy-3′-[^18^F]fluorothymidine ([^18^F]FLT) was revealed to be a promising imaging biomarker, with an excellent correlation between tumor cell proliferation rate and its uptake in lymphoma and solid tumors. More specifically, [^18^F]FLT is a PET tracer derived from the cytostatic drug azidovudine, which is intracellularly trapped after being phosphorylated by thymidine kinase-1 (TK-1). TK-1 is the initial enzyme activated during the S-phase of the cell cycle and is involved in DNA synthesis. This results in increased [^18^F]FLT uptake in highly proliferating malignant cells, characterized by enhanced DNA synthesis and upregulation of TK-1 [9,12]. That said, it is worth mentioning that this tracer is not usually significantly incorporated into DNA, accounting for less than 2% of its uptake. As a result, it doesn’t directly measure cellular proliferation but instead reflects the activity of TK-1 rather than DNA synthesis [10]. In this setting, leukemic blasts demonstrate a significant overexpression of TK-1, 10-fold higher than normal cells. Additionally, there is a notable upregulation of nucleoside transporters, specifically equilibrative nucleoside transporter 1 [ENT-1], in these cells, which enhances the intake of nucleosides, including [^18^F]FLT. Nevertheless, the precise mechanism responsible for [^18^F]FLT uptake remains unclear, and further research is required to determine the impact of membrane transporters and various nucleoside-metabolizing enzymes on this process [2]. Interestingly, it is important to note that different types of therapy may alter FLT metabolism by increasing cellular efflux and modifying TK-1 activity [11].

[^18^F]FLT PET/CT generates high-contrast images that effectively depict both lymphoma and actively proliferating tissues, demonstrating an impressive sensitivity of 97.8% [10]. Consequently, it presents a valuable option for noninvasive evaluations of proliferation rate, activity, tumor grading, and early response assessments in hematological malignancies [13,14].

This review aims to provide an overview of the existing literature on the clinical applications of [^18^F]FLT PET/CT in individuals with hematological malignancies. Additionally, when feasible, it will emphasize a comparison with [^18^F]FDG PET/CT, which presently serves as the “gold standard” tracer in this field.

## 2. Materials and Methods

A literature research was performed on PubMed/Medline, Scopus, Embase, Cochrane library, and Google Scholar databases to find any available original articles on the clinical use of [^18^F]FLT PET/CT in patients with hematological malignancies. The keywords of the inquiry, both as text and MeSH terms, variously combined, were: “hematological malignancies”; “lymphoma”; “positron emission tomography”; “PET”; “3′-deoxy-3′-[^18^F]fluorothymidine”; “FLT”. To identify supplementary, eligible articles, the references of the retrieved articles were also screened for additional papers. Two reviewers (A.G.N. and J.G.) screened, retrieved, and selected data from each manuscript. Original articles edited in the English language and performed on human patients were included in the review. Preclinical studies, including in vitro and animal models, were, on the contrary, excluded. A table with the main characteristics of the included articles has been created (PMID, first author, year of publication, type of hematological malignancy, clinical application, PET parameter, and the main findings), as shown in Table 1.

## 3. Results

The literature search retrieved 22 articles; among them, 7 were excluded after reviewing titles, abstracts, and full texts because of their preclinical nature. Finally, a total of 15 studies were selected for the analysis [1,2,8,9,10,11,13,14,15,16,17,18,19,20,21]. All studies had a prospective design. Among them, 12/15 analyzed lymphoma patients (mixed lymphoma subtypes in 8/12 and diffuse large B-cell lymphoma [DLBCL] in 4/12) [1,8,9,10,11,13,14,15,17,18,19,20], whereas 3/15 studies analyzed the role of [^18^F]FLT PET in acute myeloid leukemia (AML) [2,16,21].

Considering biodistribution in patients affected by lymphoma and comparing it to [^18^F]FDG biodistribution, a significantly higher [^18^F]FLT uptake was observed in bone marrow, liver, and spleen (*p* < 0.05). Despite these findings, the uptake in malignant lymphoma at baseline was similar for both radiotracers [9,10]. However, aggressive lymphoma exhibited a significantly higher [^18^F]FLT uptake compared to indolent forms. Applying receiver operating characteristic (ROC) analysis, mean standardized uptake value (SUVmean) distinguished between aggressive and indolent lymphoma with an area under the curve (AUC) of 0.98 (as well as FLT-SUVmax with an AUC of 0.97), whereas [^18^F]FDG SUVmean distinguished between these two forms of neoplasm with a lower AUC of 0.78 ([^18^F]FDG-SUVmax, AUC 0.79). Furthermore, it has been reported that a high SUV of 4.5 had the potential to indicate clinically aggressive and histologically high-grade lymphoma already at the initial staging, and it was suggested that a SUV cutoff of 3 could reliably differentiate between indolent and aggressive forms of lymphoma. Moreover, a correlation or a positive trend of [^18^F]FLT uptake and Ki-67 index was shown by authors [10,13].

Speaking about the prognostic role of baseline [^18^F]FLT PET/CT, Herrmann et al. in 64 DLBCL patients reported that the staging SUVmean was significantly lower for patients with complete response (CR) than for patients not achieving it (*p* = 0.049) [13]. Moreover, for both SUVmean and SUVmax, [^18^F]FLT uptake was significantly lower in the subgroup with an International Prognostic Index (IPI) score of 2 or less compared to the group of subjects with a score of more than 2 (*p* = 0.002). Conversely, Schöder et al. revealed that baseline [^18^F]FLT or [^18^F]FDG PET parameters (in particular SUVmax, [^18^F]FDG metabolic tumor volume [MTV], and [^18^F]FLT total proliferative volume [TPV]) were not associated with patient outcomes [19].

The value of interim [^18^F]FLT PET/CT (iFLT) was explored by different papers [1,11,15,17,18,19]. In a study of 92 DLBCL patients, authors reported that iFLT was the only significant independent prognosticator of 3-year progression-free survival (PFS) (hazard ratio [HR] 8.13, 95% confidence interval [CI]: 2.55–25.91, *p* < 0.0001) and 5-year PFS (HR 5.54, 95%CI: 1.97–15.60, *p* = 0.001). PFS was significantly shorter in subjects with a positive iFLT scan compared to those with a negative scan (*p* < 0.0001). Despite that, in the same cohort, the difference in PFS between the Deauville-positive and Deauville-negative patients, as well as PERCIST-positive and PERCIST-negative, was not significant [15]. In another study by Lee et al., the sensitivities and specificities of iFLT positivity in predicting disease progression or death were 88.2% and 70.5% for SUVmax and 85.7% and 66.0% for SUVmean, respectively, using cut-off values of 1.86 and 1.65 for the two semiquantitative parameters [18]. In addition, the positive predictive value (PPV) and negative predictive value (NPV) were 53.6% and 93.9% for SUVmax and 60% and 94.4% for SUVmean, respectively. When comparing the iPET results with the end-of-treatment (EOT) PET/CT results, the diagnostic performances of the interim differences of SUVmax (ΔSUVmax) and SUVmean (ΔSUVmean) were better than the values of the final ΔSUVmax and ΔSUVmean. In particular, iFLT positivity was significantly associated with a worse 5-year PFS (*p* = 0.001) and a worse 5-year overall survival (OS) rate (*p* = 0.001) compared to negative iFLT. Moreover, Herrmann et al. reported that a decrease of 79.0% for SUVmean (PPV: 92.6%) and 82.0% for SUVmax (PPV: 95.7%) at iFLT, respectively, predicted complete remission after 2 cycles of treatment [17].

The measurement of SUVmax and the percentage of its decrease after two cycles of treatment (ΔSUVFLT) were also assessed by Wang et al. and compared with the [^18^F]FDG counterpart (ΔSUVFDG) [1]. Both the [^18^F]FDG and [^18^F]FLT SUVmax values of the lesions were significantly lower after treatment than at baseline. The AUC was 0.769 for ΔSUVFDG [*p* = 0.004; 95% CI 0.615–0.923] and 0.762 for ΔSUVFLT (*p* = 0.006; 95% CI 0.605–0.918). Furthermore, a value of 79% for ΔSUVFDG achieved a sensitivity of 66.7% and a specificity of 78.6%, while a value of 76% for ΔSUVFLT achieved a sensitivity of 76.7% and a specificity of 78.6%. In this setting, both tracers and both ΔSUV cut-off values revealed significant differences in PFS and OS between low and high levels of uptake. In terms of diagnostic performance, a ΔSUVFLT change of 76% showed the highest specificity (85.7%), NPV (92.3%), and accuracy (81.8%) amongst the visual and semi-quantitative assessment parameters. The correlation with iPET and outcome was also explored by Schöder et al., who found in 65 mixed lymphoma patients that iFLT uptake evaluated by visual analysis (grade 1–3 versus grade 4–5) was a significant predictor of both PFS and OS. Conversely, iFDG residual uptake predicted PFS but not OS [19]. Furthermore, in an early therapeutic monitoring setting, it was reported that the PPV of iFLT (91%) was significantly higher than that for interim International Harmonization Project (IHP) (*p* = 0.001), EORTC (*p* = 0.001), PERCIST (*p* = 0.008), Deauville (*p* = 0.001), baseline SUVmax (*p* = 0.002), baseline total lesion glycolysis [TLG] (*p* = 0.002), baseline MTV (*p* = 0.003), interim SUVmax (*p* = 0.001), interim TLG (*p* = 0.02), interim MTV (*p* = 0.001), percentage change in SUVmax (*p* = 0.001), and percentage change in MTV (*p* = 0.003) [20].

The value of the EOT scan was evaluated by different studies [8,11,14]. It was reported that all lymphoma patients responding to chemotherapy had a significant reduction of [^18^F]FLT uptake after administration of the R-CHOP therapeutic regimen. In contrast, a high persisting uptake at the site of lymphoma manifestation was demonstrated in patients with refractory disease, with a modest decline of 39% compared with the initial uptake [11]. The study by Kasper et al. correlated both [^18^F]FDG and [^18^F]FLT PET/CT results with OS. OS was significantly longer for patients with both negative scans compared to those with [^18^F]FDG/[^18^F]FLT positivity (*p* = 0.008) [14]. Mena et al. evaluated the ability to differentiate between residual tumor and inflammation after the end of therapy with dynamic acquisition [8]. For both benign and malignant lesions, the mean time to activities curves (TACs) showed a rapid uptake of [^18^F]FLT within lymphomas, with ~90% of the activity reaching a peak at 5 to 10 min post-injection (p.i.). Moreover, it was reported that tumor uptake remained higher than that of the blood pool, with some forms of neoplasm exhibiting a continued slow uptake throughout the remaining 50 min of the dynamic scan and others showing a relative plateau after 10 min. A significant difference between the mean value of a specific parameter reflecting the available binding (λk3) between benign (0.0251 ± 0.009) and malignant lesions (0.0603 ± 0.026) (*p* = 0.01) was also reported. Single-time-point SUV analysis also showed the ability of [^18^F]FLT to distinguish between benign and malignant lesions by 30 min (*p* = 0.0083), ~1 h (*p* = 0.0003), and ~2 hours post-injection (*p* = 0.0028). Moreover, [^18^F]FDG SUVs were greater than [^18^F]FLT SUVs in lymphomas (7.8 ± 3.8 vs. 5.5 ± 2.2); however, the [^18^F]FDG uptake was often high in non-malignant tissues, resulting in false positive lesions. At ROC analyses, [^18^F]FLT estimated maximum SUV (SUVest.max) distinguished between lymphoma and inflammation with a larger AUC than [^18^F]FDG SUVest.max (0.94 ± 0.057 vs. 0.69 ± 0.12), at ~1 h post-injection. In particular, a cut-off of SUVest.max of 3.0 for [^18^F]FLT revealed a sensitivity of 90% and a specificity of 100% in predicting malignancy after treatment.

Finally, three articles [2,16,21] evaluated the role of [^18^F]FLT PET/CT in patients affected by AML, and in all of them, patients were submitted to PET imaging independently of the suspicion of the presence of myelosarcoma. In this setting, it is worthwhile to underline that usually PET imaging is not routinely performed for the assessment of this disease, reserving it for specific cases, such as the suspicion of extramedullary disease. The biodistribution study confirmed the predominant retention of [^18^F]FLT in the bone marrow and spleen, significantly higher in AML patients than in controls [2]. In contrast, the retention of [^18^F]FLT in the liver was significantly lower in AML patients compared to the controls. In the early assessment of treatment response after induction therapy, significant differences were observed between PET-positive and PET-negative groups for SUVs at all sites [21]. Moreover, patients with PET-negative findings achieved CR in the follow-up (high NPV), and patients with resistant disease (RD) showed PET-positive findings (high sensitivity). Interestingly, it was demonstrated that a reliable response assessment did not appear to be a time-dependent variation for those scans acquired during chemotherapy or shortly thereafter. However, in all the selected studies, the patient sample resulted equal to or less than 10 patients, which is a significant limitation to drawing a final conclusion on the role of [^18^F]FLT PET in AML [16].

## 4. Discussion

The thymidine analogue [^18^F]FLT clearly demonstrated its ability to reflect proliferation-dependent retention of nucleosides in hematological malignancies, which can therefore be assessed non-invasively by PET/CT [13,17]. Generally speaking, its whole-body biodistribution is favorable, displaying a high tumor-to-background ratio; however, it has limited sensitivity for evaluating bone diseases due to its high physiological uptake in the bone marrow (mean SUV value: 6.9). Furthermore, the tracer tends to exhibit less intense uptake in treated skeletal lesions compared to the surrounding normal bone marrow [9]. Similarly, its glucuronization is responsible for high physiological uptake in the liver, therefore reducing its sensitivity in the detection of liver metastases [10]. In contrast, it has been reported that [^18^F]FLT offers an advantage in the evaluation of the central nervous system by providing specific detection of lymphoma lesions, as they exhibit negligible background uptake in the brain and the skull [2,10]. Since the distribution of the tracer is different from [^18^F]FDG, the establishment of [^18^F]FLT PET/CT interpretation criteria is still an ongoing challenge.

It was demonstrated that [^18^F]FLT PET/CT showed high sensitivity in detecting aggressive lymphoma lesions and seems, therefore, a suitable tool for noninvasive assessment of tumor grading [13]. An important feature of this imaging modality is that usually the acquisition is performed from the vertex to the upper thigh after a single injection and therefore offers a non-invasive visualization and quantitative evaluation of the entire bone marrow volume. This advantage could be quite significant when assessing treatment responses compared to a single-point biopsy [16,21]. Moreover, it was underlined that aggressive lymphoma exhibits significantly higher tracer uptake compared to indolent ones, which is characterized by a heterogeneous proliferation index [10].

As has emerged, the importance of using [^18^F]FLT as a PET tracer is especially related to the predictive role of pre-therapeutic imaging and to assess therapy response. In this setting, it was underlined as a significantly lower SUVmean before treatment in patients who achieved CR. Therefore, a higher proliferation rate measured by increased [^18^F]FLT uptake is observed in patients prone to relapse or progress and represents a negative prognostic marker [13].

Recently, iFDG has been validated as a prognostic tool in patients with some hematological malignancies, however, with some limitations [18]. The introduction of [^18^F]FLT has raised expectations for improving iPET assessments, as it can reflect early cellular changes following chemotherapy administration. Furthermore, iFLT could potentially noninvasively identify high-risk patients with greater precision than iFDG, enabling individualized management [11,15,18]. In this setting, overestimation of [^18^F]FDG uptake might occur in tumors with an inflammatory component or because of chemotherapy- or radiation-mediated inflammatory processes [13]. Differently, since [^18^F]FLT is a marker of cellular proliferation, it appears as a better tracer for early response assessment after cytotoxic therapy [22]. Moreover, false-positive results have been observed when attempting to differentiate residual tumor from fibronecrotic tissue after therapy using [^18^F]FDG. Again, [^18^F]FLT uptake is closely associated with proliferation measurements, making it a more selective tracer for tumor tissue compared to [^18^F]FDG [23]. As a consequence, due to its higher specificity and PPV, [^18^F]FLT has been proposed as a tracer aimed at reducing false-positive findings [8,15,24]. Notably, iFLT emerged as a superior independent predictor of PFS compared to iFDG, using both quantitative and therapeutic assessment criteria like the Deauville or PERCIST [15]. Similarly, iFLT revealed its ability to predict both PFS and OS, with a high NPV but with insufficient PPV, which remains insufficient to justify therapy escalation without the confirmation of biopsy results [19]. Furthermore, iFLT demonstrated greater PPV and accuracy compared to the standardized interpretation criteria used for iFDG [17,19]. Nevertheless, due to the imperfect specificity of iFLT, biopsy of FLT-positive lesions should still be considered (unless there is other compelling evidence of disease) before considering a change in treatment [15].

Some studies have indicated that EOT [^18^F]FDG PET/CT scans tend to have a substantially higher PPV compared to iFDG PET scans, with similar NPV for both [17,20]. Similarly, the EOT [^18^F]FLT PET/CT demonstrated its value in the assessment of response to treatment, and it was underlined as a reliable modality to distinguish between malignant or benign lesions, with a reported sensitivity of 90% and a specificity of 100% based on both the kinetics of [^18^F]FLT uptake (as rapid tracer accumulation is observed in tumor lesion in the first 5–10 min, followed by stable tracer retention) and SUV at 1h post-injection. [8]. Interestingly, it was also reported that even earlier treatment response assessment results (as early as day 2) obtained via [^18^F]FLT PET/CT imaging correctly matched the clinical response (CR or RD on follow-up biopsy) [16]. Therefore, it might be a reasonable approach to utilize the EOT [^18^F]FDG PET/CT to compare the performance of iFLT for assessing responses in patients with hematological malignancies like DLBCL or AML [8,14,17,20].

Currently, no standardized guidelines for interpreting [^18^F]FLT PET scans, especially in terms of interim response analysis, have been established. Therefore, further research and conclusive consensus views on response analysis and the interpretation of [^18^F]FLT PET/CT data obtained from site-specific lesions are needed [18].

## 5. Conclusions

[^18^F]FLT has emerged as a promising tool for non-invasive assessment of proliferation in hematological malignancies through PET/CT imaging. The high physiological uptake of this tracer in some healthy tissues can, however, limit its use, in particular for bone diseases due to its high uptake in the bone marrow, which is higher than [^18^F]FDG. [^18^F]FLT PET/CT demonstrated the ability to differentiate between tumor and inflammation with good diagnostic accuracy, especially when compared to [^18^F]FDG, which is characterized by low positive predictive value due to its possible uptake also in non-malignant conditions. Moreover, [^18^F]FLT offers advantages in treatment response assessment in both interim and end-of-treatment evaluation, in particular for the first setting, where it seems to demonstrate a higher prognostic value compared to [^18^F]FDG.

[^18^F]FLT PET/CT has the potential for predicting patient outcomes and guiding treatment strategies. Its use in early response assessment after chemotherapy administration demonstrated its ability to identify slow and suboptimal responders with poor prognoses. Moreover, in order to obtain clear information on its role, further research and standardized interpretation criteria for [^18^F]FLT PET/CT are needed to fully harness its potential in clinical practice.

## Figures and Tables

**Table 1:** Main characteristics from the included articles.

Author, Year [Reference]	Type of Disease (No Patients)	Age (Mean ± SD or Median (Range))	Clinical Setting	Parameters	Main Findings
Wang et al., 2018 [1]	DLBCL (44 pts)	52 ± 16	Baseline, iPET (after 2 cycle), end of treatment (rituximab-based CHT) vs. [^18^F]FDG	SUVmax	iFLT PET/CT had higher accuracy than standardized [^18^F]FDG-based interpretation for therapeutic response assessment in DLBCL, reducing the number of false positive results.
Buck et al., 2008 [2]	AML (10 patients)	47 ± 13	Baseline	SUVmax	[^18^F]FLT is able to visualize extramedullary manifestation sites of AML. [^18^F]FLT uptake is also present in bone marrow, caused by both neoplastic and normal hematopoietic cells. Therefore, the correlation between [^18^F]FLT uptake in this tissue and leukemic blast infiltration did not reach statistical significance.
Mena et al., 2014 [8]	HL and NHL stage II to IV (21 pts)	46 ± 15	Therapeutic response assessment vs. [^18^F]FDG	SUVest.max; time activity curves generated from dynamic data	[^18^F]FLT PET shows improved specificity over [^18^F]FDG in distinguishing residual lymphoma from post- treatment inflammation after completing therapy.
Buchmann et al., 2004 [9]	NHL (7 pts)	48 ± 12	Radiopharmaceuticals biodistribution	SUVmax	[^18^F]FLT accumulated more intensively in aggressive NHL and NHL in transformation than in the indolent one. Organs with highest physiological uptake: bone marrow and liver.
Buck et al., 2006 [10]	Malignant lymphoma (34 pts)	51 ± 12	Staging Restaging	SUVmax SUVmean	[^18^F]FLT PET was suitable for noninvasive assessment of tumor grading. [^18^F]FLT may be a superior PET tracer for detection of malignant lymphoma in organs with high physiologic [^18^F]FDG uptake and early detection of progression to a more aggressive histology or potential transformation.
Hermann et al., 2007 [11]	High-grade NHL (22 pts)	59 ± 14	Baseline, interim, and end-of-treatment response evaluation (R-CHOP/CHOP)	SUVmax	Administration of R-CHOP/CHOP is associated with an early decrease in lymphoma [^18^F]FLT uptake. There was no reduction of [^18^F]FLT uptake after rituximab alone, indicating no early antiproliferative effect of immunotherapy.
Hermann et al., 2011 [13]	DLBCL, follicular lymphoma grade I and grade IIIB, large cell anaplastic T-cell lymphoma (66 pts)	59 ± 15	Baseline Response to R-CHOP	SUVmean SUVmax	High [^18^F]FLT uptake at baseline is a negative predictor of response to R-CHOP treatment in aggressive B-NHL and correlates with the IPI score.
Kasper et al., 2007 [14]	HL and NHL with residual m asses >2 cm (48 pts)	46 (17–76)	Therapy response assessment	SUVmax	Although [^18^F]FDG detected more lesions than [^18^F]FLT, the additional biological characterization of tumor tissue with respect to proliferation by [^18^F]FLT might be useful by providing complementary information for the iden tification of recurrence.
Minamimoto et al., 2021 [15]	DLBCL (92 pts)	59 ± 15	Interim response evaluation after two cycles R- CHOP or R-EPOCH vs. [^18^F]F-FDG	SUVmax	In patients with DLBCL given R-CHOP/R-EPOCH, iFLT PET/CT is a superior independent predictor of outcome compared to iFDG PET/CT.
Vanderhoek et al., 2011 [16]	AML (8 pts)	48 ± 19	Different time points during therapy	None	[^18^F]FLT PET imaging during induction chemotherapy may serve as an early biomarker of treatment response in AML.
Hermann et al., 2014 [17]	DLBCL (54 pts)	62 (26–80)	Baseline and interim evaluation (one week after the start of R-CHOP)	SUVmax, SUVmean	iFLT showed relevant discriminative ability in predicting CR. Very early [^18^F]FLT PET in the course of R-CHOP is feasible and enables identification of patients at risk for treatment failure.
Lee et al., 2014 [18]	High-grade NHL (61 pts)	57 (29–80)	Baseline, interim PET (after 1 cycle), end of treatment evaluation	SUVmax, SUVmean	iFLT PET is a predictor of PFS and OS. Early [^18^F]FLT PET imaging also has a potential to identify patients with delayed response and non-favorable prognosis.
Schöder et al., 2016 [19]	Advanced-stage B-cell lymphoma (65 pts)	55 (21–71)	Baseline, interim (after 1 or 2 cycle), end of treatment (R-CHOP based chemotherapy)	Visually (using a 5- point score) or semi-quantitatively (using TPV, SUVmax and ΔSUV)	[^18^F]FLT PET after 1–2 cycles of chem- otherapy predicts PFS and OS, and a negative iFLT may potentially help design risk-adapted therapies in patients with aggressive lymphomas. In contrast, PPV of iFLT PET remains too low to justify changes in patient management.
Minamimoto et al., 2016 [20]	DLBCL (60 patients)	59 ± 13	Interim and end of treatment vs. [^18^F]F-FDG	Visual interpretation	Early iFLT PET/CT had a significantly higher PPV than standardized [^18^F]FDG PET/CT-based interpretation for therapeutic response assessment in DLBCL
Han et al., 2017 [21]	AML (10 patients)	53 ± 17	Post-induction therapy assessment	SUV	[^18^F]FLT PET/CT after induction therapy showed good sensitivity and NPV for evaluating resistant disease in patients with AML.

Abbreviations: DLBCL, diffuse large B-cell lymphoma; R-CHOP, Rituximab—cyclophosphamide, doxorubicin hydrochloride (hydroxydaunomycin), vincristine sulfate (Oncovin), prednisone; SUVmean, mean standardized uptake value; SUVmax, maximum standardized uptake value; IPI score, International Prognostic Index score; [^18^F]FLT, 3′-deoxy-3′-[^18^F]fluorothymidine; B-NHL, B-cell non-Hodgkin lymphoma; HL, Hodgkin lymphoma; NHL, non-Hodgkin lymphoma; [^18^F]F-FDG, fluorine-^18^ fluorodeoxyglucose; SUVest.max, estimated maximum standardized uptake value; R-EPOCH, rituximab, etoposide, prednisone, vincristine, cyclophosphamide, doxorubicin; iFLT-PET/CT, interim [^18^F]FLT PET/CT; iFDG-PET/CT, interim [^18^F]FDG PET/CT; PFS, progression-free survival; OS, overall survival; PPV, positive predictive value; CR, complete response; AML, acute myeloid leukemia; NPV, negative predictive value; SD: standard deviation.

## Data Availability

Data supporting the reported results can be found using the public PubMed/MEDLINE, Scopus, and Google Scholar databases.
